# An automatic approach for classification and categorisation of lip morphological traits

**DOI:** 10.1371/journal.pone.0221197

**Published:** 2019-10-29

**Authors:** Hawraa H. Abbas, Yulia Hicks, Alexei Zhurov, David Marshall, Peter Claes, Caryl Wilson-Nagrani, Stephen Richmond

**Affiliations:** 1 School of Engineering, Kerbala University, Kerbala, Iraq; 2 School of Engineering, Cardiff University, Cardiff, Wales, United Kingdom; 3 School of Computer Science and Informatics, Cardiff University, Cardiff, Wales, United Kingdom; 4 School of Dentistry, Cardiff University, Cardiff, Wales, United Kingdom; 5 Medical Imaging Research Center, University of Leuven, Leuven, Belgium; Ohio State University, UNITED STATES

## Abstract

Classification of facial traits (e.g., lip shape) is an important area of medical research, for example, in determining associations between lip traits and genetic variants which may lead to a cleft lip. In clinical situations, classification of facial traits is usually performed subjectively directly on the individual or recorded later from a three-dimensional image, which is time consuming and prone to operator errors. The present study proposes, for the first time, an automatic approach for the classification and categorisation of lip area traits. Our approach uses novel three-dimensional geometric features based on surface curvatures measured along geodesic paths between anthropometric landmarks. Different combinations of geodesic features are analysed and compared. The effect of automatically identified categories on the face is visualised using a partial least squares method. The method was applied to the classification and categorisation of six lip shape traits (philtrum, Cupid’s bow, lip contours, lip-chin, and lower lip tone) in a large sample of 4747 faces of normal British Western European descents. The proposed method demonstrates correct automatic classification rate of up to 90%.

## Introduction

**Fig 1:**
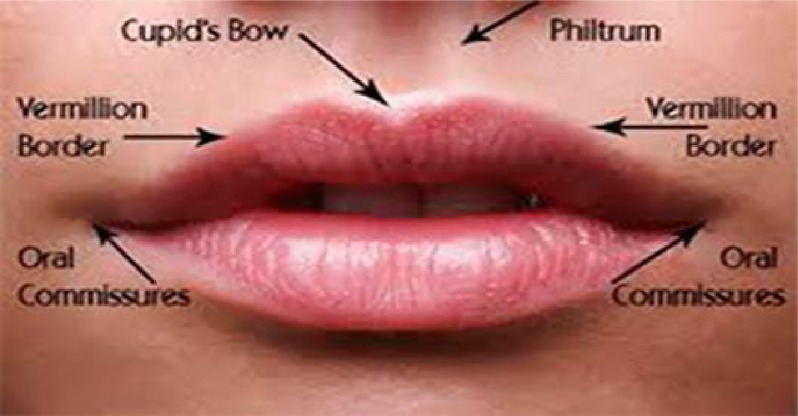
Basic morphological lip features.

The face is the most expressive part of the human body and is essential in everyday social interaction. The lips are one of the key components of the face (Fig 1); the lip area runs from the base of the nose to the tip of the chin, and therefore constitutes most of the lower third of the face. The lips include the philtrum and Cupid’s bow. The lip vermillion is the thin layer of skin, red in colour, overlying a highly vascularized region. The appearance of the lips varies with facial movement; therefore, for accurate anthropometric measurement the lips should be assessed when the subject is relaxed and has a natural head posture [1]. The morphological features of the lips vary greatly between individuals and are particularly dependent on age, sex and ethnicity [2, 3].

Studying lip morphology is important for many applications, including face recognition and gender classification [4–7]. The lips have also been reported to contribute to facial attractiveness and a number of studies have attempted to evaluate lip aesthetics by creating norms and standards of ideal lip position and shape based on cephalometric analysis and facial measurements [8–11]. Furthermore, lip morphology plays an important role in diagnosis and analysis of many medical conditions and facial dysmorphology. It is also important to find genetic variants associated with facial syndromes.

Cleft lip (CL) is one of the most recognisable facial anomalies which has been the focus of clinical research for many decades [12]. The association between genetic variants and environmental factors associated with cleft lip has also been reported [13]. Fetal alcohol syndrome (FAS) is another medical condition where lip morphology is used in a diagnostic role. FAS features are smooth philtrum, thin upper lip vermilion and short palpebral fissure length [14].

**Fig 2:**
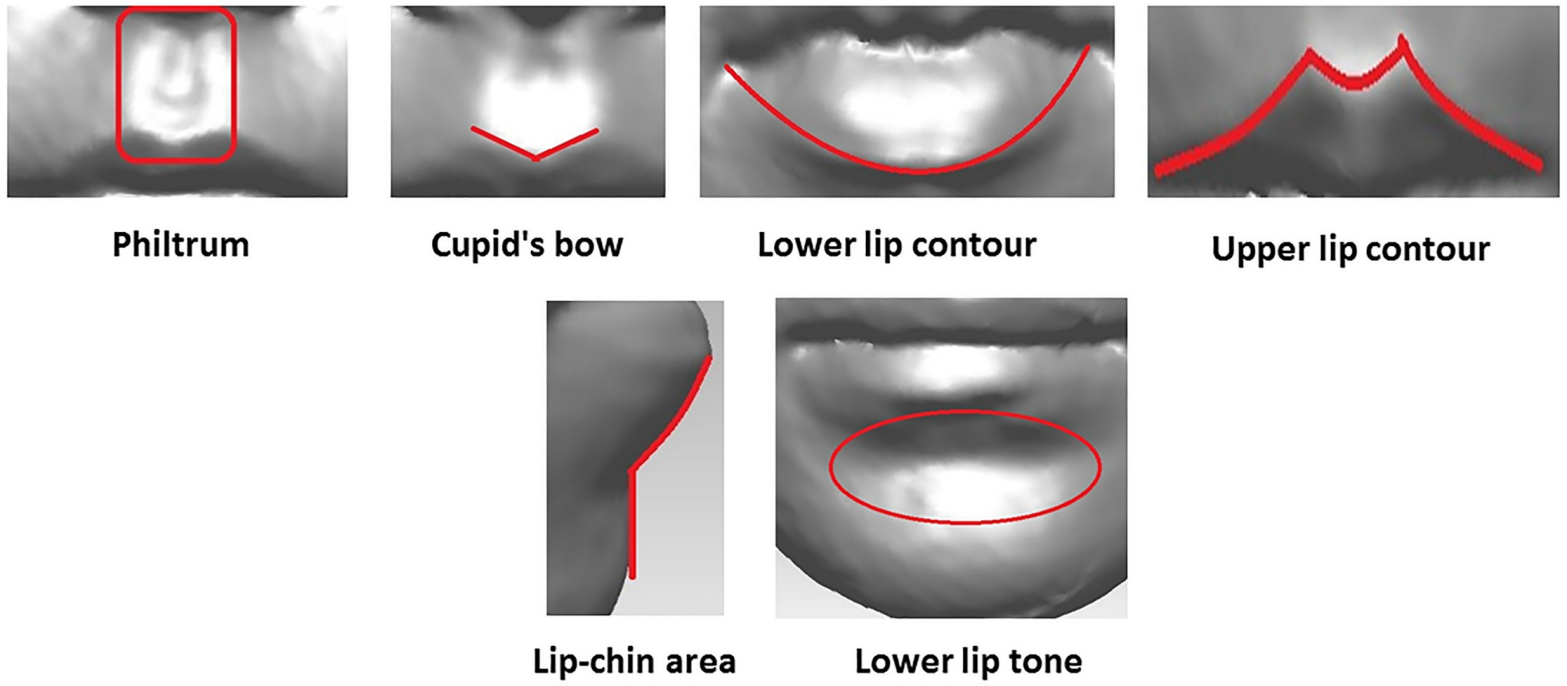
Lip traits. Description of the lip traits used in the present study.

Thus, the identification and classification of lip shape characteristics is important in medical practice. So far, the categorisation (clustering) of lip morphological characteristics (traits) has mostly been performed subjectively in clinical practice, which is time consuming and prone to error. In this paper, we propose a method for automatic classification and categorisation of lip morphological traits. We investigate six shape traits of the lip area (Philtrum, Cupid’s bow, lip contours, chin, and lower lip tone) using the proposed approach (Fig 2). These traits were categorised subjectively by medical experts and enables a direct comparison with an automated approach.

The proposed method relies on a combination of 3D geometric features, which exhibited robust results for gender classification in our previous study [15]. These features are derived from the mean and Gaussian local curvatures, shape indices, and curvedness measures obtained for *geodesic path* (shortest path) between anthropometric landmarks in the lip area.

The main contributions of this work can be summarized as follows:

- It proposes an approach for automatic categorisation and classification of lip morphology. To the best of our knowledge, no such automatic approach has been suggested so far.
- It employs novel geodesic curvature features for the description of lip shape.
- It adopts a new method, based on partial least squares regression, for visualising the effect of the trait categories on the lip region.
- It compares the results of the automatic categorisation with those of the subjective(manual) categorisation previously reported in [16]. The supervised classification rates based on the automatic categories outperform those based on the manual categories by at least 8%. In addition, the automatic labels were found to be more efficient than the manual labels.

## Related work

The traditional methods that have been employed to assess and categorise lips morphological traits can be divided into three categories: clinical examination, ordinary two-dimensional (2D) photography, and three-dimensional (3D) facial imaging.

In clinical examination, certain features (e.g., lip width, height) are analysed for the subject by direct observation or measurement performed by a medical expert [17, 18]. This method is accurate to some degree but need specific instruments and trained expert clinician to make the measurements.

Ordinary 2D photography is the most common method of recording human’s facial morphology due to the lost cost, ease of use and availability of the necessary equipment. For example, in the past, 2D photographs of faces were used to obtain anthropometric measurements and classification of lip morphological traits [19, 20]. However, 2D photographs do not capture 3D face shape, which contains important morphological information.

In the last two decades, 3D imaging (e.g. 3D meshes obtained using laser or other imaging technologies) has become more common in various medical applications. 3D facial images are much more informative than 2D photographs [21, 22] and thus can be more useful for studying lip morphology. For example, Wildon et al. [23] used 3D facial images of 109 subjects, aged 5–6 years, to produce four categories for philtrum shape: triangular, parallel, concave, and flat. Later on, Wilson et al. [16] produced 3D measurements of lip vermilion and Cupid’s bow and described different morphological features of the vermilion of the lips and associated lip traits for 4747 subjects from the ALSPAC dataset [16]. In Addition, Lee et al. [24] used the 3D face to identify measures of facial appearance for designing reconstructive surgery for Cleft lip with or without cleft palate of Hispanic/Latino White children. Although past research indicates the popularity of 3D imaging for research in facial morphology, current methods in the area rely on solely manual facial trait classification and categorisation, which is a very time consuming process. Consequently, such research would benefit significantly from an automatic approach.

In the data mining world, automatic categorisation (clustering) and classification are two types of learning methods. Both these methods characterise objects into groups based on one or more features.The key difference between categorisation and classification is that categorisation is an unsupervised learning technique used to group similar instances on the basis of features whereas classification is a supervised learning technique used to assign predefined tags to instances on the basis of features [25]. Generally, the performance of the clustering or classification algorithm depends heavily on the nature of processed dataset [26, 27]. In this paper, Support Vector Machine (SVM) [28] and boosting algorthim [29] are used for automatic classification and Kmeans++ [30] for automatic categorisation.

The majority of previous studies in facial morphology used simple geometric features such as the Euclidean distances and angles between landmarks [31, 32]. Nevertheless, 3D facial meshes contain far richer information related to the 3D shape of the faces. Therefore, new informative surface shape features are employed in this paper for the classification and categorisation of lips morphological traits. These features are derived from the mean and Gaussian curvatures, shape indices, and curvedness measures calculated at certain points along the *geodesic path* between 3D facial anthropometric landmarks. The mean, max and min curvatures have been successfully utilised to classify philtrum morphology in the past [33]. The shape index and curvedness measures have been successfully applied in a variety of 3D face recognition applications [34–37]. In addition, the geodesic paths have been widely used in face recognition (FR) systems for faces with different poses and expressions (e.g., see [38–40]). The above studies employed the radial geodesic paths or iso-geodesic paths of the whole face as features for FR purposes. In the present study, the geodesic paths are identified between the anthropometric 3D face landmarks (see Section 3.3.1).

Many studies have used partial least squares (PLS) regression [41] for analysing the effects and determine the statistical significance of biological and environmental variables such as age, gender, ethic and BMI on face module [42–44]. Consequently, we adopted PLSR with dummy variables for visualising the influence of the discovered categories on the facial physical appearance to gain insight into suitability of categories for description of the underlying facial traits.

## Materials and methods

### Dataset, landmarks and manual categorisation of lip traits

**Fig 3:**
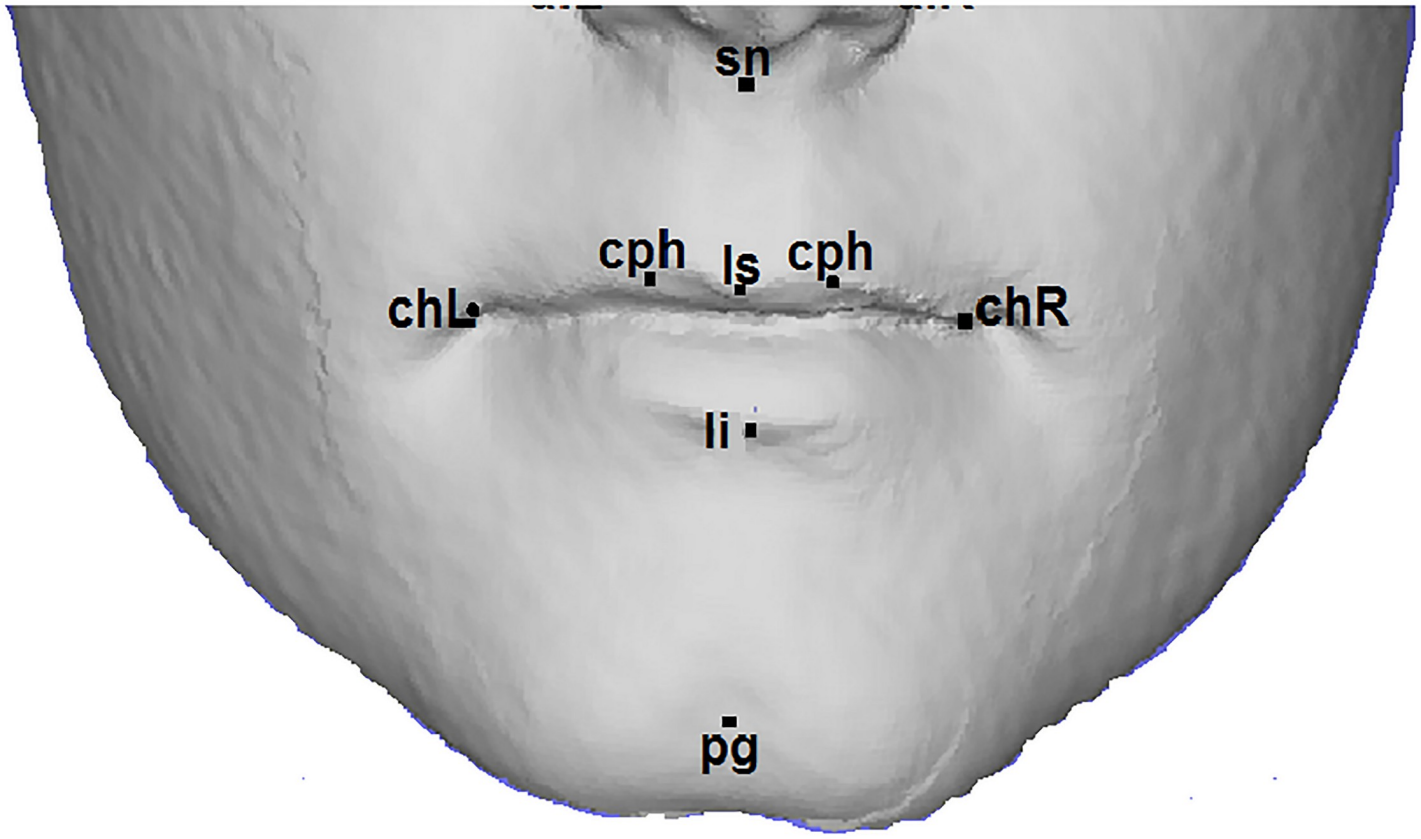
Lip landmarks. There are eight anthropometric landmarks localized in the lip region.

This study is based on three-dimensional facial data collected from 15-year-old children from the Avon Longitudinal Study of Parents and Children [45]. Ethical approval for the study was obtained from the ALSPAC Ethics and Law Committee and the Local Research Ethics Committees (UBHT): 06/Q2006/531 Avon Longitudinal Study of Parents and Children (ALSPAC), Hands on Assessments: Teen Focus 3 (Focus 15+) (7th August 2006;Confirmed 15th September 2006). Written consent was also obtained from parents and guardians prior to obtaining the facial scans. This prospective study recruited pregnant women living in the former county of Avon in South-West England with an estimated delivery date of between April 1st 1991 and December 31st 1992. The initial number of pregnancies enrolled was 14,541 (for these at least one questionnaire was returned or a “Children in Focus” clinic had been attended by 19/07/99). Of these initial pregnancies, there were a total of 14,676 fetuses, resulting in 14,062 live births and 13,988 children who were alive at 1 year of age. Please note that the study website contains details of all the data that is available through a fully searchable data dictionaryand variable search tool REF (http://www.bris.ac.uk/alspac/researchers/data-access/data-dictionary/) The children were invited to a research clinic when they were 15 years old. A subset of 4,747 children (2,233 males, 2,514 females) attended this clinic and had a three-dimensional facial scan taken using two Konica Minolta Vivid 900 laser cameras [46]. The reliability of image capture has been reported extensively elsewhere [47]. After that, 21 facial landmarks represented by a total of 63 (*x*, *y*, and *z*) coordinate values were manually identified (Manually obtained landmarks were already available for this dataset. However, they can be obtained automatically using one of the methods described in [48–50].) and recorded for each 3D facial image [47]. The biological landmark points for the lip region on the human face are illustrated in Fig 3. Table 1 explains the definitions of the landmarks. S1 Table records the 21 facial landmarks (*x*, *y*, and *z*) values for all ALSPAC data set subjects.

**Table 1:** Biological definitions of soft tissue landmarks in the lip region.

Landmark	Definition
Subnasale (sn)	Mid-point of angle at columella base
Labiale superius (ls)	Mid-point of the upper vermilion line
Labiale inferius (li)	Mid-point of the lower vermilion line
Crista philtri (cph) L/R	Point on the left/right elevated margins of the philtrum just above VL
Cheilion(ch) L/R	Point located at left/right labial commissure
Pogonion(pg)	Most anterior midpoint of the chin

Data used for this research will be made available on request to the ALSPAC executive committee (alspac-exec@bristol.ac.uk). The ALSPAC data management plan (available here: https://proposals.epi.bristol.ac.uk) describes in detail the policy regarding data sharing, which is through a system of managed open access.

This paper deals with six lip traits previously identified in [16]. Specifically, these are philtrum shape, Cupid’s bow shape, upper and lower lip contours, lateral lip-chin shape, and lower lip tone shape (Fig 2). Each trait was manually categorised into three to seven categories depending on its appearance and the respective labels were assigned to all ALSPAC images [16].

### Proposed algorithm

In this subsection, we describe the approach we use for automatic classification and categorisation of lip morphology. To the best of our knowledge, it represents the first automatic algorithm ever developed for these purposes, while previous approaches relied on manual work of highly trained clinicians. An automated approach has the benefit of bringing technology to a wider general use and opening up a potential for analysis of large datasets with direct advantages to research areas such as finding genetic associations and automatic diagnosis of facial syndromes.

**Fig 4:**
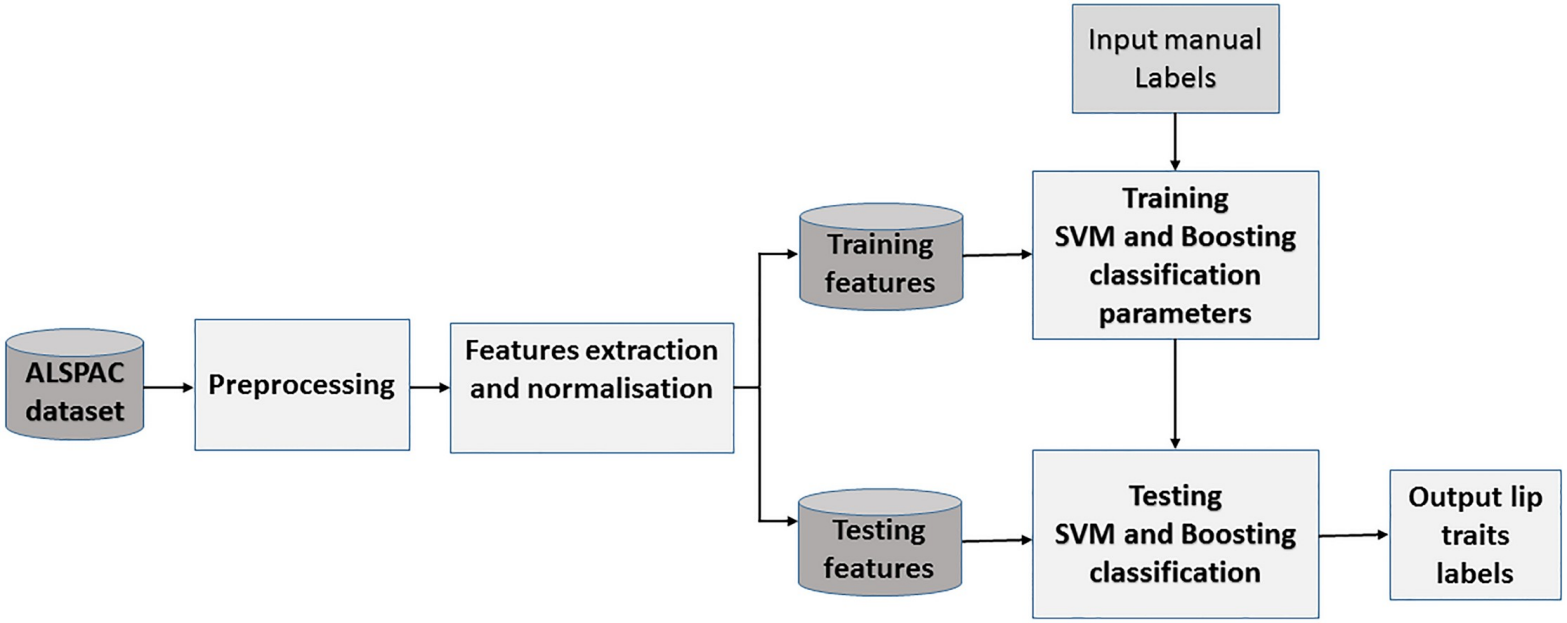
Classification block diagram. This diagram illustrates the proposed approach for automatic classification lip traits.

**Fig 5:**
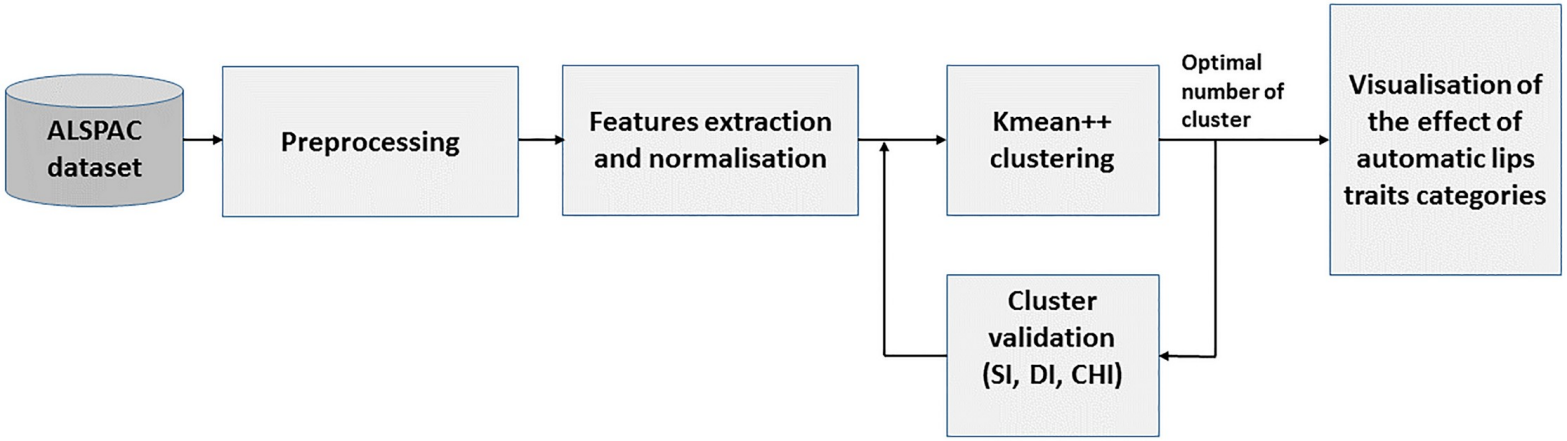
Categorisation block diagram. This diagram illustrates the proposed approach for automatic categorisation lip traits.

Figs 4 and 5 display block diagrams of the proposed approach, which are explained below.

#### Initial processing of facial images

The facial data captured by 3D scanners frequently suffers from imperfections. These imperfections are generally in the form of noise, unwanted elements (such as hair, eyelashes, ears, or portions of the neck or clothes), or holes due to missing data at dark, shiny, or hairy regions (such as eyebrows, beard, the iris, or open mouth). The removal or correction of the imperfections is an essential preliminary stage of data analysis, usually called preprocessing. In addition, the preprocessing often includes remeshing of the resulting images, to ensure that the mesh becomes more uniform and has no defects, and normalisation of the face posture, to ensure that all faces are in the same position [51]. For further analysis, a regularisation of the number and position of vertices can also be made through finding dense correspondences in the mesh [52, 53].

In this paper, two approaches for 3D face preprocessing are implemented for comparing their effects on lip area morphological traits classification, one without the regularisation and the other with the regularisation.

#### Preprocessing without mesh regularisation

All raw images were processed using the Rapidform 2006 software following the steps mentioned above [54] except for regularisation of the mesh. Specifically, the steps taken were:

- Denoising. The unwanted elements of the scans were removed manually followed by automatic removal of large spike, which can naturally arise in scanning. The images were further smoothed using the volume-preserving Laplacian filter available in Rapidform, which removes little spikes. Laplacian smoothing is a common procedure (e.g., see [54–56]), which is implemented in many software packages including, for example, Graph MATLAB Toolbox [57].
- Hole filling. Small or large holes may arise in scanning and denoising. All holes were automatically identified and filled using Rapidform built-in tools with curvature preserving option. we can also use 3DFaceModelsPreprocessingTool1 [58], to eliminate noise and remove undesired parts of the face and fill the holes automatically.
- Registration and merging. All faces were obtained using two laser cameras generating two scans of the face, left and right [59]. After denoising and hole filling, the left and right images were registered together using the Rapidform best-fit tool followed by merging to create a single facial image. The mesh quality was further checked and defects removed automatically.
- Normalisation. To insure that all faces are in the same position, all images were manually landmarked [47] and normalised by fitting them to a vertical cylinder [51], with mid-endocanthion used as the origin of coordinates.

Images obtained following these steps will be referred to as *non-regularised*.

#### Preprocessing with mesh regularisation

In addition to the above steps, all non-regularised images were superimposed on a reference face with 7150 vertices and regularised to ensure that the resulting meshes have exactly 7150 vertices each. Specifically, the following was done:

- The preprocessing steps without mesh regularisation are repeated.
- Non-rigid registration. The idea was to use an anthropometric facial template with 7150 vertices to crop the facial images of interest. The template was an average face constructed from 3D facial images of 400 Western Australian healthy young individuals, aged 5–25 years, captured with a 3dMD imaging system [53]. The template was mapped onto the facial meshes using an iterative closest point (ICP) algorithm [60].
- Regularisation. An iterative procedure was used to assign each floating point of the template to a corresponding point of the target image by applying the distance weighted k-nearest neighbor rule [61]. As a result, all faces were cropped and converted to uniformly distributed meshes each consisting of 7150 vertices.

The resulting images will be referred to as *regularised*.

It must be noted that the non-regularised images were of much higher resolution (50 to 70 thousand vertices each) than the regularised images (exactly 7150 vertices each). The regularised images were further used to test the robustness of our 3D geometric features as well as the classification and categorisation systems on low-resolution meshes in comparison with the non-regularised images.

**Fig 6:**
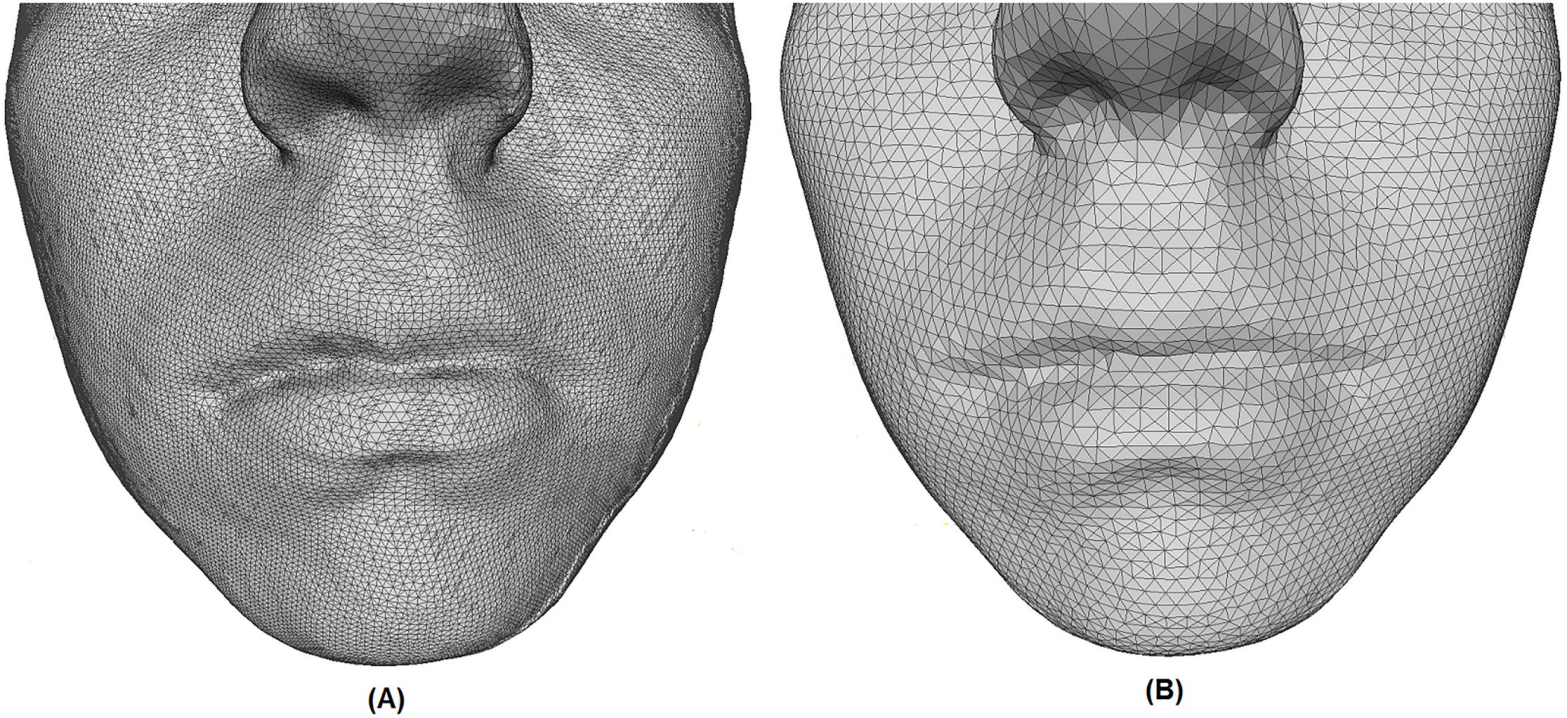
Difference between preprocessed images. (A) Non-regularised face. (B) Regularised face.

Fig 6 illustrates the difference between an non-regularised and a regularised face. It is apparent that the lip morphology is more prominent in non-regularised faces. However, regularised images allow for faster extraction of facial features. Furthermore, the regularisation allows us to test our automatic classification method for both high-resolution images (e.g., captured with laser scanners) and low-resolution images (e.g., obtained using structured light stereo or photogrammetric scanners).

#### Feature extraction and normalisation

Previous studies have shown advantages of using curvatures in 3D facial applications. This prompted us to use curvatures to classify and cluster facial morphological traits, too; however, we adopted a different, novel strategy. Specifically we decided to use *geodesic paths* between anthropometric landmarks to define key points at which curvatures can be calculated and combine these curvatures into feature descriptors for the anatomical facial traits in the lip region (Cupid’s bow shape, lip contours, etc.).

We also calculated the Euclidean and geodesic distances to assess their efficiency for the classification and categorisation of lip traits as compared to combinations of geodesic curvature features. This is because many previous studies frequently used these quantities as features for 3D facial morphology analysis (e.g., see [31, 62, 63]).

#### Extracting geodesic paths

**Fig 7:**
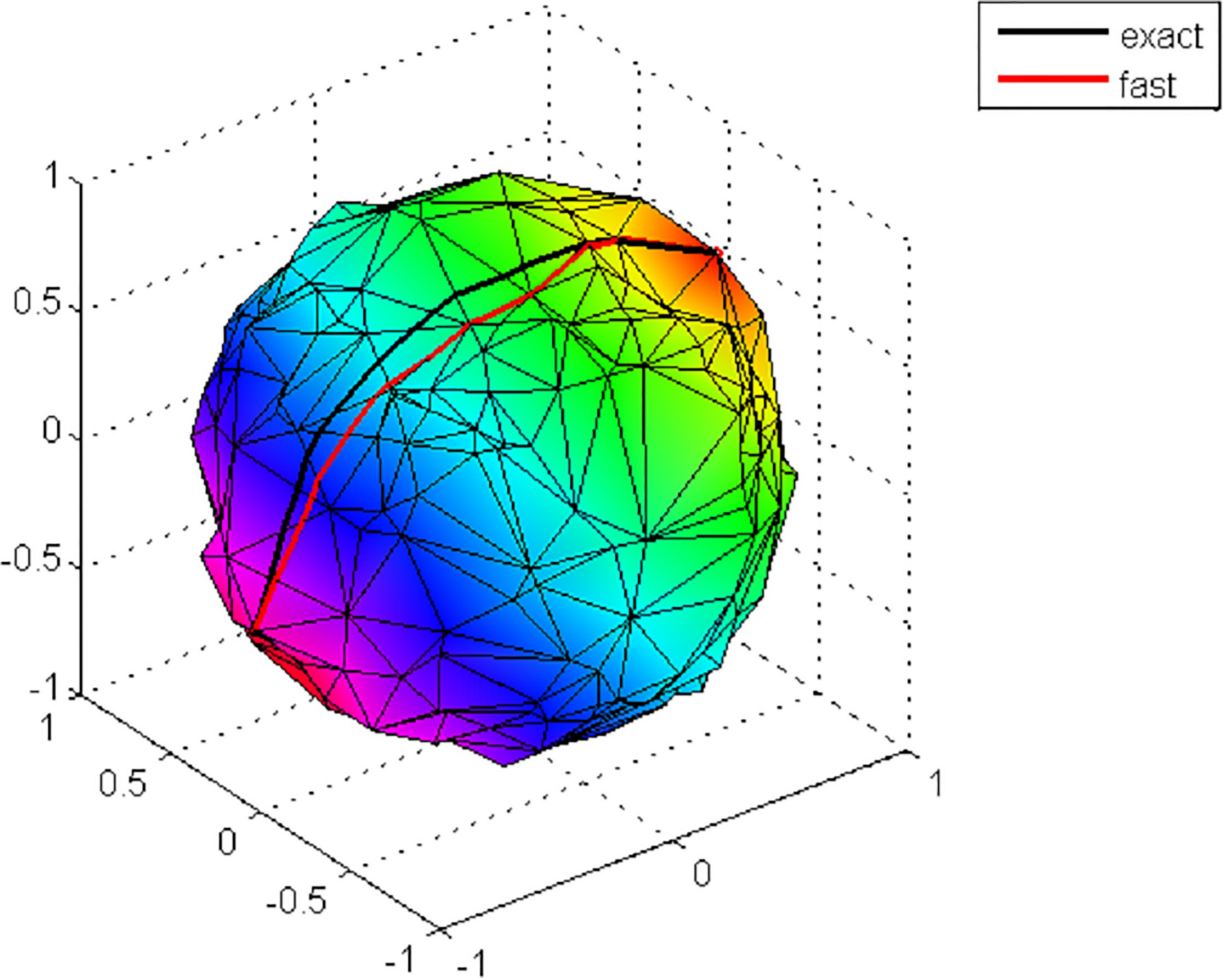
Exact versus fast marching algorithm. Extracting geodesic paths using an exact and a fast method for a synthetic mesh.

The geodesic path is the shortest route between two points on a surface and the *geodesic distance* is the length of this route [64]. There are a number of algorithms to compute geodesic paths and distances on triangular meshes; some are approximate, such as the fast marching method [65], while others are exact (however, relatively slow). The exact algorithms include, for example, the Mitchell–Mount–Papadimitriou (MMP) [66], Chen–Han (CH) [67] methods and parallel fast marching algorithm [68]. Exact methods are more accurate in tracking geodesic paths than approximate methods and are especially advantageous for low-resolution meshes. Fig 7 highlights the difference between an exact and a fast geodesic algorithm in determining geodesics in a synthetic low-resolution mesh, where the black (exact) trajectory clearly follows the mesh edges more regularly than the red (fast) trajectory.

Gabriel Peyre’s MATLAB fast-marching toolbox [69] and the exact geodesic toolbox [70] were used to find geodesic paths and calculate the geodesic distances between two landmarks. The former toolbox was used for high-resolution data (non-regularised meshes), while the latter one was used for low-resolution data (regularised meshes), because, as shown in Fig 7, the exact method is more suitable for tracking low-resolution meshes and this is compatible with the finding of [71].

**Fig 8:**
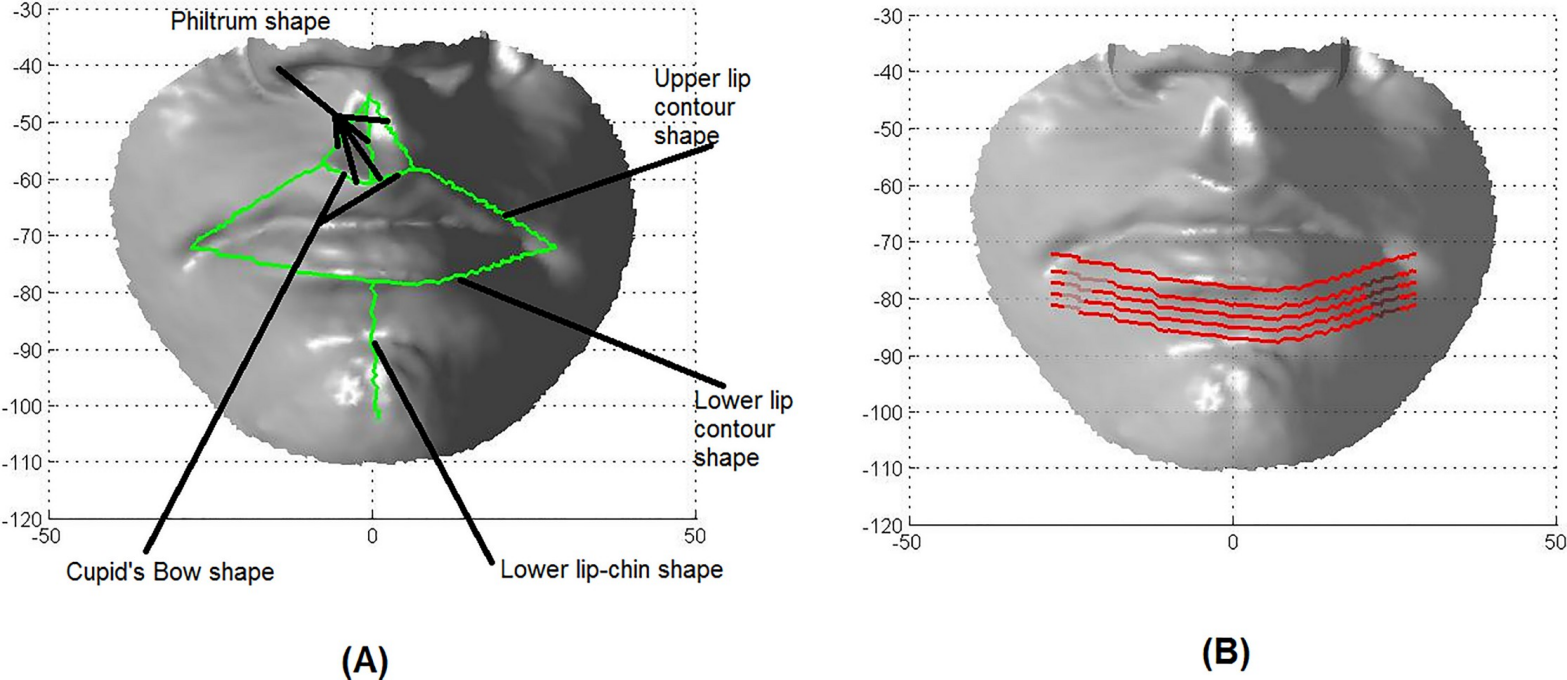
The geodesic paths used for the classification and categorisation of the lip traits. (A) Paths for the lip traits. (B) Paths for the lower lip tone.

Fig 8A illustrates the paths used for all lip traits (see Table 2), apart from the lower lip tone trait. For the lower lip tone, we used the geodesic path between the lower lip contour landmarks (chL, li and chR) and four extra geodesic paths connecting the three points obtained from the above landmarks by shifting them down by a certain distance (Fig 8B).

**Table 2:** List of geodesic paths defining morphological lip traits.

Trait name	List of related geodesic paths
Philtrum shape	sn-cphL, sn-ls, sn-chpR, cphL-ls, cphR-ls
Upper lip contour	chL-cphL, cphL-ls, ls-cphR, chpR-clR
Cupid’s bow	cphL-ls, ls-cphR
Lower lip contour	chL-li, li-chR
Lip-chin area	li-pg

where sn, cphL, ls, chpR, chL, chR, li, and pg are the lip region landmarks (see Fig 3)

#### Curvature features

The local principal curvatures were first calculated at the vertices of the geodesic path. The mean curvature, Gaussian curvature, shape index, and curvedness were then calculated from the principal curvatures.

The principal curvatures result from the intersection of the 3D surface with a orthogonal to the tangential plane. Many methods have developed to calculate the principal curvatures for the 3D images [72, 73]. In this work the *Normal Cycle* curvature tensor method is used to calculate the principal curvatures for the 3D images. This method was implemented, in particular, by [74] to give a general method to define principal curvatures for the 3D images. The principal curvatures *k*_1_ and *k*_2_ at *v* are estimated by the eigenvalues of *normal cycle theory based equation* T(v), while the eigenvectors represent the curvature directions [75].

$$\begin{array}{c} T\left(v\right)=\frac{1}{\left|B\right|}\sum_{\text{edges}}\beta \left(e\right)|e\cap B|\;ee^{T} \end{array}$$ (1)

where *v* represents the vertex position on the mesh, |*B*| is the surface area around *v* over which the curvature tensor is estimated, *β*(*e*) is the signed angle between the normal vector to the two oriented triangles incident to edge *e*, |*e* ∩ *B*| is the length of *e* ∩ *B*, and *e* is a unit vector in the same direction as *e* [75].

Using principal curvatures (*k*_1_ and *k*_2_), Mean (*M*), Gaussian (*Ga*) curvature, Shape index (*Sh*) and curvedness (*C*) are calculated as:

$$\begin{array}{c} M=\frac{k_{1}+k_{2}}{2} \end{array}$$ (2)

$$\begin{array}{c} Ga=k_{1}.k_{2} \end{array}$$ (3)

$$\begin{array}{c} Sh=0.5-\frac{1}{\pi }.tan^{-1}(\frac{k_{1}+k_{2}}{k_{1}-k_{2}}) \end{array}$$ (4)

$$\begin{array}{c} C=\sqrt{\frac{{(k_{1}}^{2}+{k_{2}}^{2})}{2}} \end{array}$$ (5)

Shape index (*Sh*) quantitatively measures the shape of a surface at a vertex *v* and captures the intuitive notion of local shape of a surface. while, curvedness (*C*), measures how highly or softly bent a surface is. Curvedness can define the scale difference between objects: for example the difference between a soccer ball and a cricket ball. See [33] and [76] for more information on the principal curvature calculation algorithm.

#### Calculation of the geodesic and Euclidean distances

For each lip trait, the geodesic distances and Euclidean distances were calculated between the same landmarks as those utilised to extract the geodesic paths shown in Fig 8a. The fast marching algorithm and exact algorithm were used to compute geodesic distances from high-resolution and low-resolution faces, respectively. The Euclidean distance $D_{\;AB}$ between points *A* and *B* in three dimensions, defined by their coordinates (*X*_*A*_, *Y*_*A*_, *Z*_*A*_) and (*X*_*B*_, *Y*_*B*_, *Z*_*B*_), is calculated as

$$\begin{array}{c} D_{\;AB}=\sqrt{{\left(X_{B}-X_{A}\right)}^{2}+{\left(Y_{B}-Y_{A}\right)}^{2}+{\left(Z_{B}-Z_{A}\right)}^{2}} \end{array}$$ (6)

#### Normalisation of the curvature features

Histogram normalisation is used to normalize the features descriptors. It is an estimate of the probability distribution of a continuous variable. It is a kind of bar graph, to construct a histogram, the first step is to “bin” the range of values—that is, divide the entire range of values into a series of intervals—and then count how many values fall into each interval. The bins are usually specified as consecutive, non-overlapping intervals of a variable. If the bins are of equal size, a rectangle is erected over the bin with height proportional to the frequency—the number of cases in each bin. A histogram may also be normalized to display “relative” frequencies. It then shows the proportion of cases that fall into each of several categories, with the sum of the heights equaling one [77]. In this work, each geodesic path has a different number of nodes (vertices) for curvature calculation. To deal with this, a normalised histogram distribution was calculated for each path feature; for this purpose, the number of bins selected was 5, 10, 15, 20, or 25, depending on the minimum number of nodes in a path across the entire sample, the node numbers for the longest path (li-pg) were around 100 node. The histogram representation has used for features normalisation and representation in many image and pattern recognition such as Bag-of-Words (BoW) image representation [78], this motivate us to use the histogram representation to normalise the geodesic path curvature features.

The number of vertices defining a geodesic path varies between faces. A normalisation procedure is required to ensure that the respective paths of all facial images have the same number of points at which curvature features are measured. To this end, a histogram distribution was calculated for each feature; the number of bins selected was 5, 10, 15, 20, or 25, depending on the maximum and minimum number of points in the path.

Let $P_{1}^{k},\;\ldots \;,\;P_{n}^{k}$ denote the vertices of a path *P*^*k*^ on facial mesh *k* and let $M_{i}^{k}$, $Ga_{i}^{k}$, $C_{i}^{k}$, and $Sh_{i}^{k}$ denote, respectively, the mean curvature, Gaussian curvature, curvedness value, and shape index value evaluated at vertex $P_{i}^{k}\;(i\;=\;1\;,\ldots \;,\;n)$. For each path, we choose a number *b* = 5, 10, 15, 20, or 25 such that *b* ≤ min *n*, where min *n* is the minimum number of vertices in all paths *P*^*k*^ across the sample. After the histogram normalisation (using the MATLAB function histnorm) with *b* bins, we get exactly 4*b* characteristic curvature features for path *P*^*k*^:

$$M^{k}\;=\;\left[{\hat{M}}_{1}^{k},\ldots ,{\hat{M}}_{b}^{k}\right]$$ (7)

$$Ga^{k}\;=\;\left[\hat{G}a_{1}^{k},\ldots ,\hat{G}a_{b}^{k}\right]$$ (8)

$$C^{k}\;=\;\left[{\hat{C}}_{1}^{k},\ldots ,{\hat{C}}_{b}^{k}\right]$$ (9)

$$sh^{k}\;=\;\left[\hat{S}h_{1}^{k},\ldots ,\hat{S}h_{b}^{k}\right]$$ (10)

where ^ denotes the respective values resulting from the histogram normalisation. Then a features descriptor is composed **D**^*k*^ = [**M**^*k*^, **Ga**^*k*^, **C**^*k*^, **Sh**^*k*^] consisting of 4*b* components. This procedure provided us with four vectors of equal length: mean curvature, Gaussian curvature, shape index, and curvedness. These vectors were then concatenated to produce a single vector, feature descriptor, for each path in a face; see Fig 8 and Table 2.

#### Data balancing and classification

The problem of imbalanced datasets can arise in classification when the number of elements in one class is much lower than that in other classes. Standard classifiers tend to overestimate the importance of the larger classes and underestimate the importance of the smaller classes. To cope with this problem, several methods have been suggested [79–81].

The present study uses the boosting method [29] to classify the unbalanced ALSPAC dataset. We also compare our results with those obtained using the multiclass SVM (supper vector machine) method [28], which does not involve data balancing. In this work, the public MATLAB Software called *Multiclass Gentle Adaboosting* was used for classification purposes [82].

#### Automatic categorisation

The present study carries out automatic categorisation of lip traits by using the Kmeans++ clustering technique [30, 83]. Kmeans++ function on the same rule of kmeans for data clustering by choosing *K* initial centroids, where *K* is a user specified parameter. kmeans algorithm concepts is very simple, it is work on minimizing the sum of squared distances from a cluster center, to the cluster members.

The kmeans algorithm proceeds major steps are:

- initial centroids *K* is selected randomly.
- The group of points are assigned to nearest cluster according to Euclidean distance metric.
- The centroids to the mean of the members of the cluster is updated.
- The second and the third steps are repeated until the assignments from the second step do not change [30].

Kmeans++ handle the problem of choosing the cluster center randomly by performing a procedure to initialize the cluster centers before implementing the standard kmeans optimization iterations. The Kmeans++ initialization steps are:

- First centroid is chosen randomly.
- the distance for each data point to the nearest centroid that has already been chosen is computed.
- the next centroid is selected using a weighted probability proportional to distance value.

Using the Kmeans++ initialization procedure provides more accurate cluster centroid with minimum time [83].

To evaluate the clustering performance and find an optimum number of clusters, we used three different internal validation indices: silhouette index (SI), Dunn index (DI), and Calinski–Harabasz index (CH) [84, 85]. Internal validity indices rely on properties intrinsic to the data set. Most index measures are based on the concept that the points in the same cluster should be similar and the points in different clusters should be dissimilar. Below we briefly discuss how these concepts are defined for each of the three internal index measures:

- The silhouette index (SI) validates the clustering performance based on the pairwise difference of between-cluster and within-cluster distances. Moreover, the optimal cluster number is determined by maximizing the value of this index. This index, denoted *s*(*i*), is computed as
  $$\begin{array}{c} s\left(i\right)=\frac{\left(b(i)-a(i)\right)}{\max\{a(i),b(i)\}} \end{array}$$ (11)
  where *a*(*i*) is the average distance between the *i*th element and all other elements within the same cluster, while *b*(*i*) is the minimum average distance between the element *i*th and any other cluster, of which the *i*th element is not a member [84, 86].
- The Calinski–Harabasz index (CH) evaluates the cluster validity based on the average between-cluster and within-cluster sum of squares. CH index calculates separation based on the maximum distance between cluster centroids, and measures compactness(Compactness measures how closely the objects in a cluster are related [86]) depending on the sum of distances between objects and their cluster center. In addition, the optimal cluster number is determined by maximizing the value of this index. This index is computed as
  $$\begin{array}{c} \text{CH}=\frac{\text{trace}(S_{\text{B}})}{\text{trace}(S_{\text{W}})}\;\frac{n_{p}-1}{n_{p}-k} \end{array}$$ (12)
  where *S*_B_ is the between-cluster scatter matrix, *S*_W_ is the internal (within-cluster) scatter matrix, *n*_*p*_ is the number of clustered samples, and *k* is the number of clusters [84, 85].
- The Dunn index (DI) measures the minimum pairwise distance between objects in different clusters as the inter-cluster separation and the maximum diameter among all clusters as the intra-cluster compactness. The optimal cluster number isdetermined by maximizing the value of this index. To calculate the Dunn index, one has first to compute the distances between all the data points as follows:
  $$\begin{array}{c} \text{DI}=\min_{1\leq i\leq c}\{\min\{\frac{d(c_{i},c_{j})}{\max_{1\leq k\leq c}\left(d\left(X_{k}\right)\right)}\}\} \end{array}$$ (13)
  where *d*(*c*_*i*_, *c*_*j*_) defines the inter-cluster distance between clusters *X*_*i*_ and *X*_*j*_, *d*(*X*_*k*_) is the intra-cluster distance of cluster (*X*_*k*_), and *c* is the number of clusters [84–86].

#### Visualisation using partial least squares regression

Finally, we wish to analyse the relationship between automatically or manually determined trait categories and the geometric characteristics of the corresponding facial region. A common approach to establishing relationships in data is regression. The main difficulty in using dense data is the large number of correlated dependent variables in comparison to the number of observations, leading to model instability when using the linear least squares regression. We have addressed this problem by using the more advanced technique of partial least squares regression (PLSR) [41]

The aim of partial least squares regression is to establish a linear relationship between two sets of variables, *X* and *Y*, where *X* is the set of dependent variables and *Y* is the set of independent variables. In our case, *Y* is the set of dummy variables defining the manual or automatic categories, while *X* is the set of the *x*, *y*, and *z* coordinates of all vertices in a regularised facial mesh.

However, whilst categorical variables with two values may be directly entered as predictor or predicted variables in a multiple regression model, categorical variables with more than two values cannot be entered directly into a regression model. Therefore, we will use *dummy variables* [87]. A dummy variable is an artificial variable created to represent an attribute with two or more discrete values rather than continuous values as in standard regression. Therefore, dummy variables are created in such situations to force the regression algorithm to analyse variables correctly.

We converted the categorical variables (labels) into dummy variables [87]. For *C* categories, we need to *C* − 1 dummy variables before starting the regression process to determine their multiple and partial effects on the lip region.

The effects of trait categories on lip morphology can be illustrated using color maps. The regression coefficients define a set of weights at the vertices of the facial mesh. These weights define the magnitude and the direction of the vertex displacement per unit of the predictor (the predictors here are the label’s dummy variables). The values of interest represented in the heat maps are: (1) the ‘partial coefficients’ (magnitude); (2) the proportion of the variance at each vertex explained (partial *R*^2^) by the predictor; and (3) the degree of significance of the effect at each vertex [43, 44].

#### Statistical analysis

In this research, analysis of variance (ANOVA) statistical method is used to test differences between two or more model or methods. ANOVA is a statistical analysis strategy for extraordinary tastefulness, utility and flexibility. It is the most effective technique accessible for breaking down the data from tests. ANOVA computer software is widely used for experimental tests, it is used to test general differences among model or methods [88]. Analysis of variance (ANOVA) is used to determine if the means of two or more groups are markedly different from each other. ANOVA checks the influence of one or more factors by comparing the means of different samples. The basic terminologies of ANOVA are:

- Grand Mean: Mean is the average of a range of values. There are two types of means that are used in ANOVA test calculations, these are separate sample means and the grand mean. The grand mean is the mean of all observations combined.
- Hypothesis: It is an educated guess about something in the world around us. It should be testable either by experiment or observation. ANOVA uses a Null hypothesis and an Alternate hypothesis. The Null hypothesis in ANOVA is correct if all the sample means are equal, or when they do not have significant difference. In contrast, the alternate hypothesis is correct if at least one of the sample means is different from the rest of the sample means.
- Between Group Variability: It points to variations between the distributions of individual groups as the values within each group are different.
- Within Group Variability: It points to variations caused by differences within individual groups. In other words, no interactions between group samples are considered [89, 90].

The Analysis of variance (ANOVA) test is simple applied test and it is used dramatically in many research for statistical analysis specialty in medical application and clinical diagnosis [89, 91, 92]. Therefore, in this research the ANOVA test is used to statistically analysis the effectiveness of geodesic curvature feature in lips morphology classification and categorisation.

## Results

Six computational experiments were designed in order to assess the performance of proposed approaches for lip traits classification and categorisation. Experiments 1, 2, 3 and 4 were designed to investigate the best features to classify lip traits using the manual labels provided in [16]. In these experiments, the classification performance is measured using classification accuracy and AUC (Area Under ROC Curve) values. The ROC curve and AUC value are commonly used for evaluation local feature descriptors in both 2D and 3D images, for example in [93, 94]. That k-fold cross validation is a statistical method used to estimate the performance of machine learning algorithms.The choice of k is usually 5 or 10, but there is no formal rule. As k gets larger, the difference between the training set size and the testing subsets gets smaller [95].

In this work, The accuracy and AUC values are the average results for 5-folds (4745 ALSPAC face meshes were used in the classification task) cross validation runs. In Experiment 5, the proposed automatic approach for lip traits categorisation is assessed. Finally, in Experiment 6, the efficiency of the manual and automatic categorisation are compared using visualisation method. A detailed explanation of these experiments is provided in the following subsections.

### Experiment 1: Classification based on 3D Euclidean distances

Euclidean distances are commonly used to study face morphology [31, 96, 97]. In this experiment, we used the inter-landmark Euclidean distances shown in Fig 8A to classify the lip traits. We combined several Euclidean distances defining a lip trait to form a classification descriptor; for example, five distances were combined together to classify the philtrum shape. Table 3 lists the accuracies as well as the AUC values for the classification performed using SVM and boosting classifiers for all lip traits except for the lower lip tone. Both the non-regularised and regularised meshes were considered. The accuracies suggest that Euclidean distance is a poor measure for the classification of lip traits. However, the results are approximately the same for both the non-regularised and regularised images, which indicates that Euclidean distance is not very sensitive to mesh resolution. S2 Table represents the Euclidean distance between the lips landmarks in Table 1.

**Table 3:** Classification results based on Euclidean distance.

Lip traits	Non-regularised mesh		Regularised mesh	
	SVM	boosting	SVM	boosting
	**Accuracy**			
Philtrum shape	56.7	**62**	56	60.9
Cupid’s bow	56	**62.7**	55.4	62.5
Upper lip vermilion contour	56.6	**60**	57	58.9
Lower lip vermilion contour	57	**61.5**	56	60.7
Lower lip-chin shape	55	**60.8**	55.5	60.2
	**AUC values**			
Philtrum shape	0.558	0.628	0.552	0.615
Cupid’s bow	0.566	0.631	0.560	0.622
Upper lip vermilion contour	0.570	0.620	0.577	0.610
Lower lip vermilion contour	0.567	0.605	0.548	0.610
Lower lip-chin shape	0.553	0.603	0.549	0.600

This table lists the classification accuracies and AUC values. The classification was performed using the SVM and boosting methods for the non-regularised and regularised meshes.

### Experiment 2: Classification based on 3D geodesic distances

In the second experiment, we used geodesic distances to classify the lip traits. Many studies (e.g., see [98, 99]) suggest that geodesic distances could describe 3D models better than Euclidean distances. We calculated the geodesic distances using the fast marching and exact geodesic algorithms; these distances were between landmarks in the lip region, as shown in Fig 8A. Table 4 lists the classification results obtained with these features using, as above, two classification methods (SVM and boosting) for both the non-regularised and regularised meshes. The accuracies were slightly higher as compared to the results obtained using the respective Euclidean distances. The results also show little sensitivity to mesh resolution, since the exact method was utilised for much coarser, regularised meshes, thus providing higher performance than the fast marching method. S3 Table represents the geodesic distance between the lips landmarks in Table 1.

**Table 4:** Classification results based on geodesic distance.

Lip traits	non-regularised mesh		Regularised mesh	
	SVM	boosting	SVM	boosting
	**Accuracy**			
Philtrum shape	61.8	**66.9**	61	65.7
Cupid’s bow	60.9	**65.6**	60.2	64.5
Upper lip vermilion contour	59.6	**65**	58.7	63
Lower lip vermilion contour	59.9	**65.6**	58.6	64
Lower lip-chin shape	62	**67.3**	61	65.6
	**AUC values**			
Philtrum shape	0.614	0.668	0.61	0.653
Cupid’s bow	0.595	0.664	0.595	0.643
Upper lip vermilion contour	0.604	0.667	0.608	0.650
Lower lip vermilion contour	0.605	0.672	0.609	0.630
Lower lip-chin shape	0.608	0.659	0.608	0.654

This table lists the classification accuracies and AUC values. The classification was performed using the SVM and boosting methods for the non-regularised and regularised meshes.

### Experiment 3: Classification based on 3D geodesic path curvatures

The previous two experiments utilised the Euclidean and geodesic distances as morphological lip features, which are traditionally used for classification. In contrast, the third experiment is based on a geodesic path curvature descriptor. This descriptor combines the mean curvature, Gaussian curvature, shape index, and curvedness calculated for the points of the geodesic path between landmarks (Fig 8A). The curvatures of all geodesic paths defining a lip trait were combined together to form a feature descriptor of this trait for the classification purposes. S4, S5, S6, S7, S8, S9, S10 and S11 Tables record the mean curvature, Gaussian curvature, shape index, and curvedness for Cupid’s bow (cphL-ls and ls-cphR paths).

The lower lip tone was the most difficult morphological trait to classify, because its geometric features had to be determined in a narrow area very close to and below the lower lip contour. To this end, we used several geodesic paths approximately parallel to the lower lip contour to cover the desired area, as shown in Fig 8B. To find the optimal number of such paths and the distance between them, tuning tests were carried out followed by comparing the classification accuracies. The best accuracy was obtained for five paths separated in the vertical direction approximately by 2 mm. Table 5 lists the classification accuracies and AUC values for two classification methods (SVM and boosting) for the non-regularised and regularised meshes. The classification accuracies increased markedly, to 71–77% for the non-regularised and 67–72% for the regularised meshes. One can see that the classification accuracies depend on mesh resolution, because the curvature features are clearly sensitive to mesh resolution, which confirms the inference made in [100].

**Table 5:** Classification results based on geodesic path curvatures.

Lip traits	non-regularised mesh		Regularised mesh	
	SVM	boosting	SVM	boosting
	**Accuracy**			
Philtrum shape	68	**74.8**	64.9	70.6
Cupid’s bow	65	**70.7**	62	67.4
Upper lip vermilion contour	66	**75.7**	62.8	69.7
Lower lip vermilion contour	66	**74**	61.8	70.6
Lower lip-chin shape	66.8	**75.5**	63.7	71.8
Lower lip tone	67	**76.8**	63.7	72
	**AUC values**			
Philtrum shape	0.665	0.759	0.640	0.712
Cupid’s bow	0.637	0.694	0.615	0.662
Upper lip vermilion contour	0.654	0.738	0.618	0.690
Lower lip vermilion contour	0.649	0.732	0.625	0.689
Lower lip-chin shape	0.653	0.750	0.655	0.705
Lower lip tone	0.675	0.756	0.647	0.714

This table lists the classification accuracies and AUC values. The classification was performed using the SVM and boosting methods for the non-regularised and regularised meshes.

### Experiment 4: Classification based on a combination of features

In this experiment, the classification performance was explored by using a combination of the Euclidean distance, geodesic distances and geodesic curvature features as a classification descriptor. The Min-Max scaling approach was used to normalise the data to a fixed range of zero to one [101].

The best performance was achieved when the geodesic distance and curvature features were combined in the classification experiment. Table 6 displays the classification results based on different combinations of features for the non-regularised and regularised faces. Using a descriptor combining the geodesic curvature features and geodesic distances increased the classification accuracy to 72–79% for the non-regularised meshes and 70–74% for the regularised meshes.

**Table 6:** Classification results based on different combinations of features.

	Combination of features					
Lip traits	GC + ED		GC + GD		GC + ED + GD	
	SVM	boosting	SVM	boosting	SVM	boosting
	**Accuracy (non-regularised faces)**					
Philtrum shape	66.8	72	69	**76.8**	69	76.5
Cupid’s bow	65	70	67	**72**	65.6	71.4
Upper lip contour	65	74.5	67	**78.5**	64.8	78
Lower lip contour	64	71.5	66	**76.4**	65.8	75.5
Lip-chin shape	64.8	73.2	69	**78**	67	76.8
	**AUC values (non-regularised faces)**					
Philtrum shape	0.655	0.725	0.664	0.770	0.680	0.772
Cupid bow	0.635	0.683	0.656	0.705	0.648	0.690
Upper lip contour	0.65	0.747	0.651	0.778	0.651	0.761
Lower lip contour	0.656	0.699	0.645	0.752	0.657	0.743
Lip-chin shape	0.640	0.723	0.690	0.759	0.665	0.757
	**Accuracy (regularised faces)**					
Philtrum shape	64.6	79.8	65.9	**72.6**	65.4	71.8
Cupid bow	61.5	65.3	63.7	**69.7**	61.9	68.8
Upper lip contour	60	69.5	63.8	**73.2**	62.8	71.7
Lower lip contour	62	70	64.8	**72.6**	64.9	72.6
Lip-chin shape	62	69.5	64.7	**74.4**	62.9	73.6
	**AUC values (regularised faces)**					
Philtrum shape	0.637	0.700	0.648	0.723	0.646	0.729
Cupid bow	0.605	0.645	0.643	0.687	0.615	0.674
Upper lip contour	0.604	0.685	0.629	0.728	0.630	0.697
Lower lip contour	0.600	0.680	0.640	0.698	0.635	0.692
Lip-chin shape	0.61	0.686	0.638	0.737	0.630	0.709

This table lists the classification accuracies and AUC values. The classification was performed using the SVM and boosting methods for the non-regularised and regularised meshes. GD stands for geodesic distances, ED for Euclidean distances, and GC for geodesic path curvature features.

### Experiment 5: Lip traits automatic categorisation

In this experiment, we used the combination of the above geodesic curvatures and the geodesic distances to categorise (cluster) the lip morphological traits without relying on manual labeling. An unsupervised clustering scheme was utilised to partition the geodesic curvature features into multiple clusters, each defined by a centroid, and the Kmeans++ algorithm was employed to perform the clustering.

**Fig 9:**
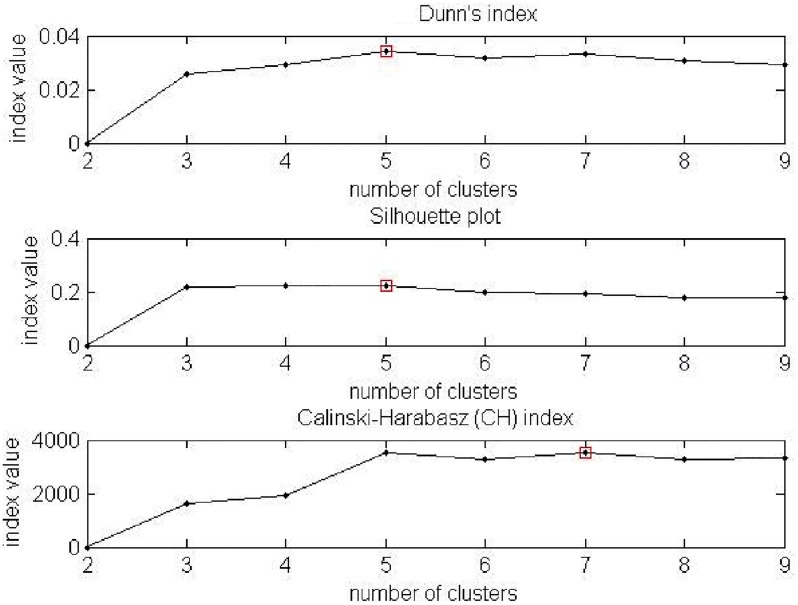
Validity indices for the number of clusters for the philtrum shape. The figure shows the CH, DI, and SI results.

Kmeans++ starts with allocation one cluster center randomly and then searches for other centers given the first one. So this algorithm uses random initialization as a starting point; hence, it can give different results on different runs. Therefore, the Kmeans++ clustering procedure was run 100 times to reduce the effect of randomisation. Out of 100 results, the clustering results that produced the minimal sum of squared distance scores was chosen. An analysis for optimum numbers of clusters was carried out using internal cluster validation techniques. We computed the validity indexes CH, DI, and SI for all traits with the number of clusters *K*, ranging from 2 to 9, with the tests repeated 50 times to find a stable number of clusters and labels. For example, Fig 9 displays the results for the philtrum shape; the optimum number of clusters is 5 (based on DI and SI) or 7 (based on CH). For the other traits, the optimum number of clusters was obtained in the same manner: 3 for Cupid’s bow, 3 or 4 for the upper lip contour, 3 or 4 for the lower lip contour, 5 for the lower lip-chin shape, and 5 for the lower lip tone shape. These results are almost the same as those produced by a medical expert in [16]. Although there are slight differences: for example, the philtrum shape was categorised subjectively into 7 clusters, while automatic categorisation produced five clusters on some runs and seven clusters on other runs. To compare with the manual results in the next experiment, the philtrum shape with 7 automatic clusters was used. Table 7 shows the percentage of the number of times the validation methods chose a certain number of clusters. While Table 8 records the number of samples in each sub class of lip traits which automatically categorised.

**Table 7:** The percentage of the number of times the validation methods chose a certain number of clusters.

	DI					SI					CH				
Traits	3	4	5	6	7	3	4	5	6	7	3	4	5	6	7
Philtrum shape	5	10	35	20	30	0	10	45	10	35	0	0	30	15	55
Cupid’s bow shape	100	0	0	0	0	100	0	0	0	0	80	20	0	0	0
Upper lip contour shape	70	30	0	0	0	45	55	0	0	0	30	70	0	0	0
Lower lip contour shape	40	60	0	0	0	55	45	0	0	0	75	25	0	0	0
Lower lip-chin shape	0	25	65	10	0	0	25	60	15	0	0	35	50	15	0
Lower lip tone shape	0	35	55	10	0	5	45	50	0	0	20	35	45	0	0

Different numbers of clusters were found to be optimum with the use of different validity indices: Dunn’s index (DI), silhouette index (SI) and Calinski–Harabasz index (CH).

**Table 8:** Prevalence of lip traits in ALSPAC dataset(%).

Trait name	class 1	class 2	class 3	class 4	class 5	class 6	class 7
Philtrum shape	3.7%	46.2%	8.8%	20.4%	4.7%	3.9%	12.3%
Cupid’s bow shape	25.6%	60.5%	13.9%				
Upper lip contour shape	35.7%	26.8%	34.0%	3.5%			
Lower lip contour shape	27.0%	43.6%	18.8%	10.6%			
Upper lip border shape	46.0%	49.4%	4.6%				
lower lip border shape	36.5%	51.0%	12.5%				
Lip-chin area	11.9%	52.3%	17.9%	14.3%	3.6%		
Lower lip Tone	40.0%	9.8%	12.9%	20.0%	7.3%		

The prevalence of lip traits using automatic categorisation. The Upper lip border shape and the lower lip border shape are the results of clustering the lip contour geodesic curvature features into three classes, S1 Fig illustrates the examiner’s rudimentary classification scale for the characterisation of lip traits.

The best classification accuracy for the manual labels was in the range of 72–79% (Table 6). The same classification procedure was repeated now with the automatic labels. Table 9 shows the classification accuracies for a descriptor combining the geodesic curvature features and geodesic distances, obtained using both manual and automatic lip morphological traits labels. In addition, the lower lip tone classification accuracy was as high as 92.7% with the automatic categorisation labels and geodesic curvatures features. However, for the manual categorisation labels and the same features, the classification accuracy for this trait was only 76.8%. It is clear that the classification based on the automatic labels *outperforms* that based on the manual labels.

**Table 9:** Classification accuracies for the manual and automatic lip area trait labels.

Trait name	Manual categories	Automatic categories
Philtrum shape	76.8%	89%
Upper lip contour	78.5%	87.6%
Upper lip border	75.5%	82.4%
Cupid’s bow	72%	84.8%
Lower lip contour	76.4%	90%
Lower lip border	72.2%	81%
Lip-chin area	78%	86%

Boosting method used to classify lip traits using the manual and automatic labels.

### Experiment 6: Visualisation of the effect of trait categories on the lip region

PLS regression was used to characterise the effects of the trait categories on the regularised 3D faces. All statistical significance tests were based on 1000 permutations [102]. The partial effects (one variable is independent of the others) in the multivariate regression were coded by the partial regression coefficients. These coefficients define label weights at the mesh vertices, which were visualised as a heat map; cooler colours correspond to weaker effects on vertices, while warmer colours correspond to stronger effects.

**Fig 10:**
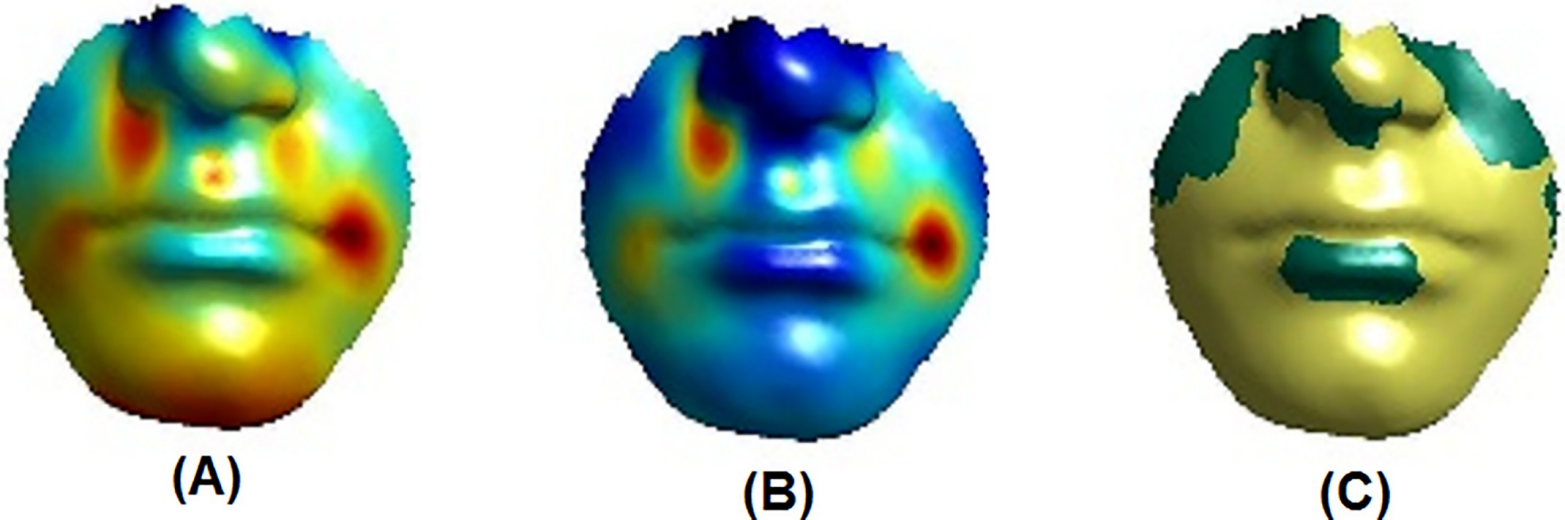
Regression results. Effect of a manual label (philtrum shape) on the lower face. (A) Partial coefficients. (B) *R*^2^. (C) *p* < 0.001 (yellow), *p* ≥ 0.001 (green). Warmer colours correspond to stronger effects.

**Fig 11:**
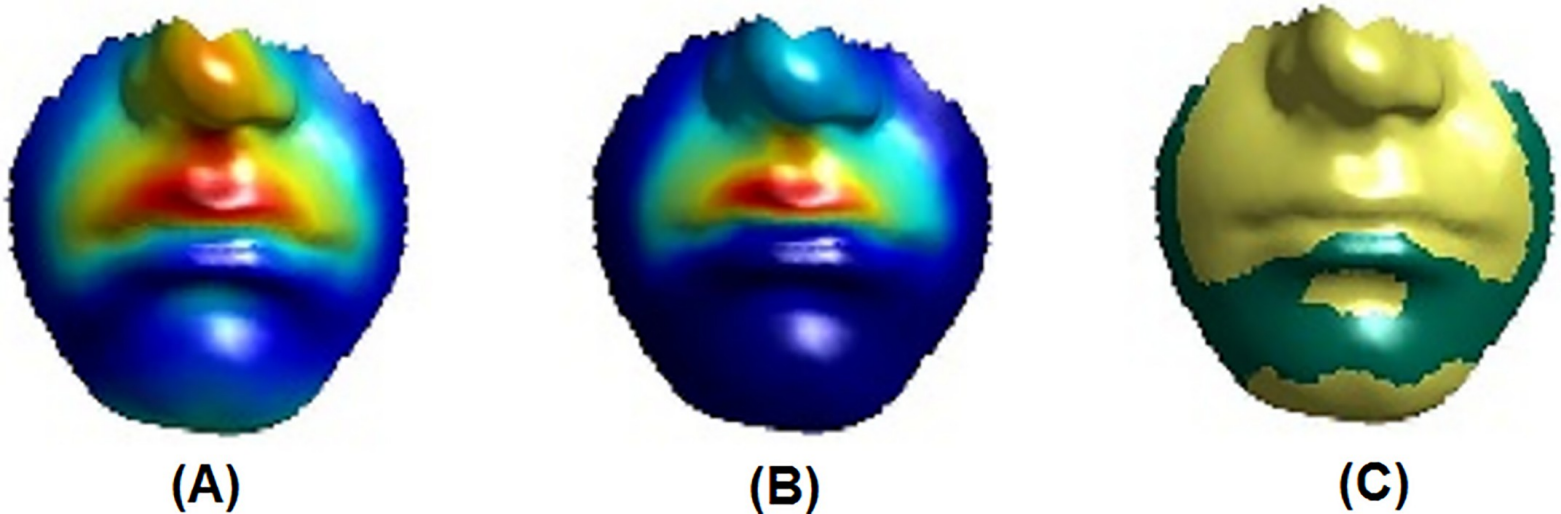
Regression results. Effect of an automatic label (philtrum shape) on the lower face. (A) Partial coefficients. (B) *R*^2^. (C) *p* < 0.001 (yellow), *p* ≥ 0.001 (green). Warmer colours correspond to stronger effects.

As an example, Fig 10 illustrates the regression results for the manual philtrum shape labels, while Fig 11 shows those for the automatic philtrum shape labels. In these figures, the “partial coefficients” correspond to the magnitude of the vertex displacement in three dimensions; the proportion of variance that the predictor variable predicts at each individual vertex is defined by partial *R*^2^. The effect of the labels was displayed as colour maps of statistical significance using two colours, with yellow indicating highly significant results (p-value < 0.001) and green showing moderately significant or insignificant results (p-value ≥ 0.001).

**Fig 12:**
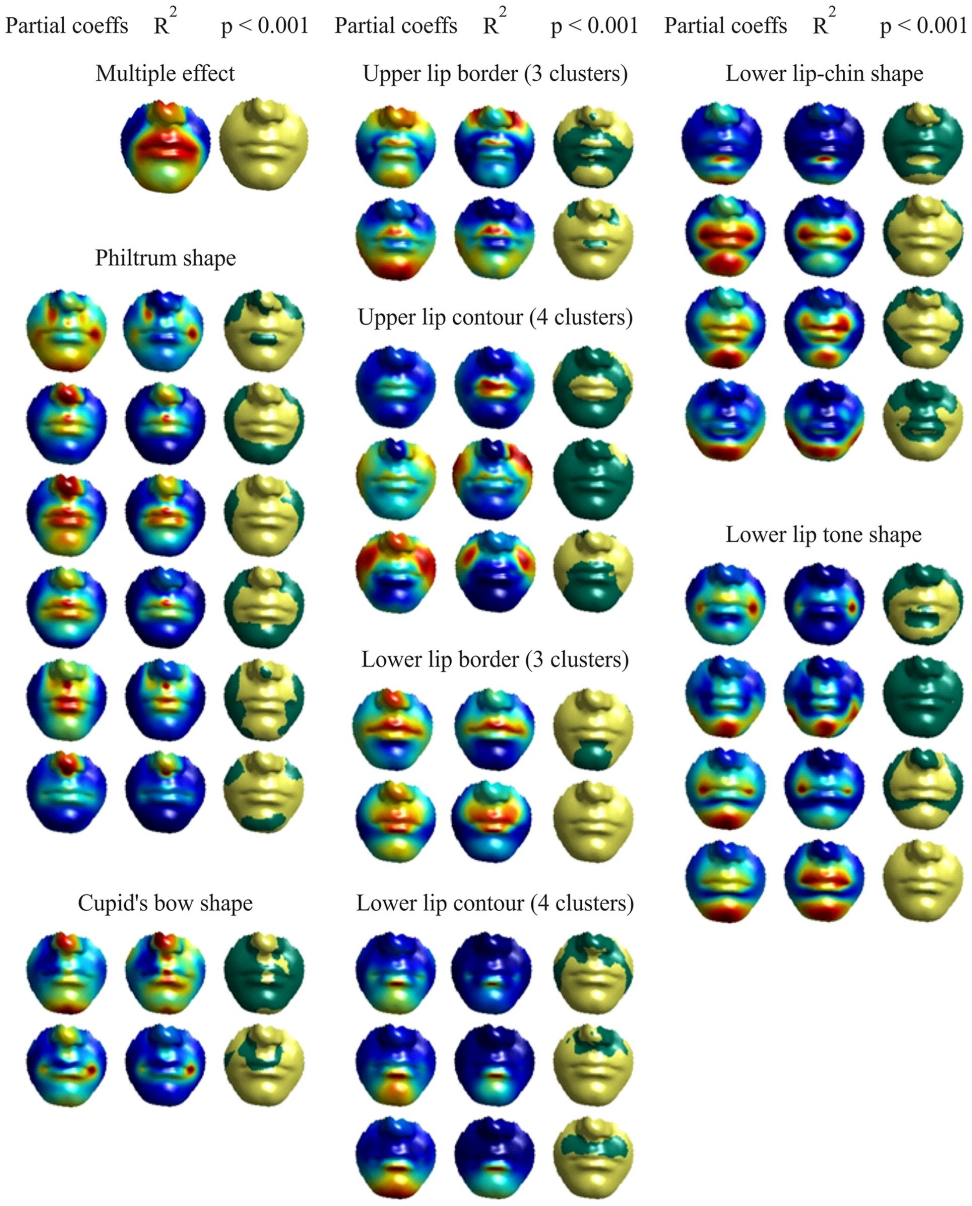
Visualisation of the effect of manual categories on the lower face based on the regression results for dummy variables. The ‘Partial coeffs’ columns display heat maps of the partial regression coefficients associated with mesh vertices (warmer colours correspond to stronger effects). The ‘*R*^2^’ columns display heat maps of proportion of the variance. The ‘*p* < 0.001’ columns show two-colour maps of the statistical significance of the effect: yellow for p-value < 0.001 and green for p-value ≥ 0.001.

**Fig 13:**
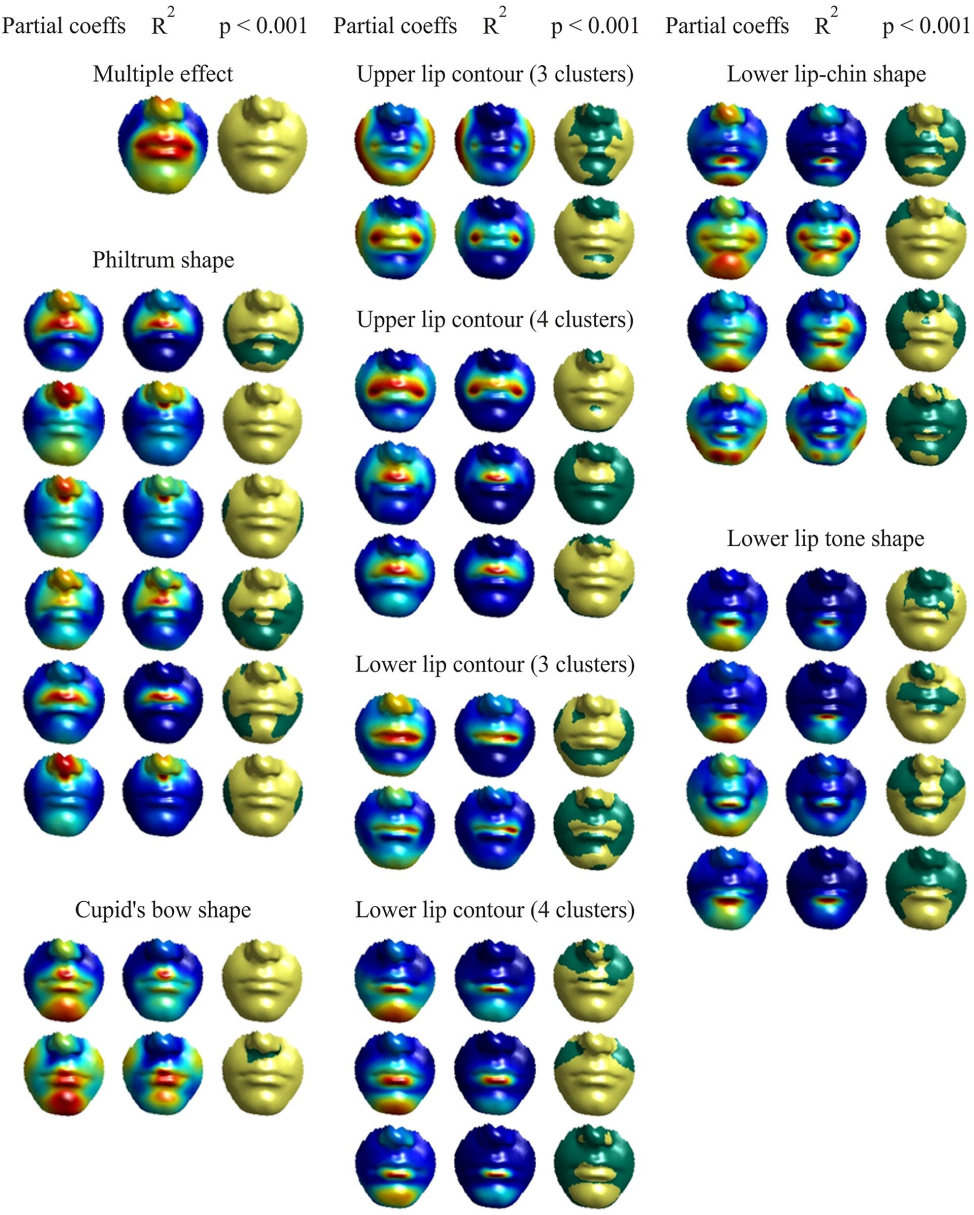
Visualisation of the effect of automatic categories on the lower face based on the regression results for dummy variables. The ‘Partial coeffs’ columns display heat maps of the partial regression coefficients associated with mesh vertices (warmer colours correspond to stronger effects). The ‘*R*^2^’ columns display heat maps of proportion of the variance. The ‘*p* < 0.001’ columns show two-colour maps of the statistical significance of the effect: yellow for p-value < 0.001 and green for p-value ≥ 0.001.

Figs 12 and 13 visualise the effects for all trait’s dummy variables as individual and multiple (the combination of all trait’s dummy variables). As can be seen from the visualisation, the multiple effect of all trait categories is concentrated and significant in the lip region.

### ANOVA test results

To determine the significance of classification improvement using the geodesic curvature features over the geodesic distance and euclidean distance features, the results of ten runs of the Boosting classifier using these three types of geometric features (Euclidean distance, geodesic distance and geodesic curvature features) are submitted to Analysis of Variance (ANOVA) statistical testing. Table 10 shows the ANOVA results, where P-value is the probability of the improvement to occur by chance, and MS is the mean square error.The F-value is a ratio Between group variability to Within group variability. The improvement is significant if the P-value is less than 0.05, which means that the improvement is unlikely to happen by chance. As the P-value is almost zero in Geodesic curvature features cases, the improvement of the proposed features over the geodesic distance and Euclidean distance features is significant.

**Table 10:** ANOVA test.

Feature type	F	p-Value	MS
Geodesic distance against Euclidean distance	2261.47	2.22E-10	210.07
Geodesic curvature features against Geodesic distance	3206.57	7.43E-20	269.48
Geodesic curvature features against features combination	3400.67	6.88E-21	300.56

The test is conducted using 10 classification accuracies. Features combination mean geodesic distance and geodesic curvature features.

## Discussion

The first three experiments in the present study aimed to determine which facial features were the most effective in automatic classification of the lip traits using non-regularised and regularised 3D meshes. Although fairly effective for both high and low resolution data, the Euclidean distances alone did not produce good enough classification accuracies. Experiment 2 showed that the geodesic distances between landmarks provided higher classification accuracies. We attribute this to geodesic distances being more informative than Euclidean distances in describing facial surfaces; this compatible with many state of the art research such as [103, 104]. The geodesic distance features classification accuracies are higher than Euclideann distance features classification accuracies and with only small differences between the non-regularised and regularised data. Experiment 3 used 3D geometric curvatures of the shortest geodesic path between two anthropometric landmarks as features in the classification experiments. The accuracies were found to improve markedly, which is likely due to the curvature features taking into account local facial geometry and, hence, characterising the lip shapes much better than the Euclidean and geodesic distances.

Most of the previous studies [33, 98, 105] have focused on using combinations of facial features to achieve higher classification accuracies. In the present study, we also adopted this approach and used a combination of Euclidean and geodesic distances with *geodesic curvature features* (see Table 6) to produce feature descriptors for the lip morphology classification. The highest accuracy was obtained when geodesic distances were combined with geodesic curvatures. The SVM (Support Vector Machine) method failed to classify the lip traits efficiently, in contrast to the boosting method, because the data were highly imbalanced in more than one class; this finding seems to corroborate the inference made in [106]. In spite of using different types and combination of features to classify the lip traits, the classification accuracies were not very high for the manual labels provided in [16]. This encouraged us to categorise the lip traits automatically. The Kmeans++ algorithm has shown good categorisation results in our experiments, which, however, does not prevent us from trying alternative clustering techniques, such as spectral clustering [107], or using distance measures to cluster the elements of classes [108] in the future. Three internal validation techniques were used to select an optimum number of clusters for each lip trait. The selection process was repeated 50 times to find stable numbers of clusters. For the lower and upper lip contours, these techniques were found to show quite contradicting results for three and four clusters. For this reason, we used the labeling results for both alternatives in Experiment 6.

Table 10 records the Analysis of Variance (ANOVA) test results. As the P-value is almost zero in Geodesic curvature features cases, the improvement of the proposed features over the geodesic distance and Euclidean distance features is significant. Using the combination features (geodesic distance and geodesic curvature features) provide smallest p-value but this value is not far from the p-value in geodesic curvature features case. From that, the ability of geodesic curvature feature in lips morpholoy is proved.

In Experiment 6, a new method was used to visualise the automatic and subjective (manual) categorisation (labeling) regression results for the lip area. All previous studies [42, 44], and [43] dealt with continuous variables. By contrast, the present study seems to be the first to deal with discrete variables (automatic or subjective (manual) labels) for face morphology classification. Using dummy variables is a way to deal with the discrete variable problem; for example, six dummy variables were used to represent the philtrum shape labels which fall into seven classes. Figs 10 and 11 show the regression results for the philtrum shape trait with one dummy variable.

Fig 12 details the regression results for the manual labels showing the multivariate effect and contribution of each individual dummy variable. Fig 13 details the regression results for the automatic labels.

On careful examination of the results, one can see that our automatic labels (categories) are fairly similar to the subjective labels (categories) [16]. However, for three out of seven traits (the lower lip-chin, the lower lip contour and lower lip tone shape), our automatic labels provide much better categorisation results, as the label effect appears at the right areas (compare Figs 12 and 13). In particular, for the lower lip tone, the subjective labels show a strong effect (red colours) near the oral commissures rather than in the mentolabial sulcus area, which is under the lower lip contour. The analysis does not give us a clear indication of the optimum number of clusters for the classification of the upper and lower lip contours. However, it is apparent from Fig 13 that using four clusters would be preferable in both cases as the colour maps for respective dummy variables highlight the correct areas better. The classification accuracy using the automatic categories has outperformed the manual categories classification accuracy by at least 8%. All this testifies that the approach for automatic categorisation of 3D facial morphology proposed in this study has a considerable potential,this categorisation technique and geodesic curvature features can be used for different facial trait to save clinicians some manual labor and produce accurate facial morphological traits categorisation results in a short time comparing to manual work.

## Conclusion

We proposed a new automatic approach to classify and categorise various facial morphological traits, with a specific application to morphological traits in the lip region, using 3D geometric features based on geodesic path curvatures. We evaluated our approach on the large ALSPAC dataset consisting of 4747 3D scans of a 15-year-old population. Six experiments were conducted to evaluate our classification and categorisation approach and to compare manual and automatic trait categories on the lip region models using the PLSR method.

In general, the geodesic curvature features provided higher classification accuracies as compared to the Euclidean and geodesic distances. The classification accuracies increased when both geodesic distance and geodesic curvature were used. The same features were used to categorise lip traits automatically, with categorisation approach based on the Kmeans++ and internal cluster validation algorithms. The results of Experiment 6 illustrate that the automatic categories are more accurate in defining lip traits as compared to the manual categories.

In future, we are planning to extend our approach to other morphological facial features such as the nose. The proposed approach may be have potential for gaining knowledge about genotype and facial traits associations, which will also be considered in future work.

## Supporting information

S1 Table Landmarks coordinates of ALSPAC dataset. (XLSX)

S2 Table Lip Euclidean distances. (XLS)

S3 Table Lip geodesic distances. (XLS)

S4 Table Path (cphL-is) curvedness features. (XLS)

S5 Table Path (cphL-is) Gaussian features. (XLS)

S6 Table Path (cphL-is) mean features. (XLS)

S7 Table Path (cphL-is) shape features. (XLS)

S8 Table Path (cphR-is) curvedness features. (XLS)

S9 Table Path (cphR-is) Gaussian features. (XLS)

S10 Table Path (cphR-is) mean features. (XLS)

S11 Table Path (cphR-is) shape features. (XLS)

S12 Table The automatic categorisation results for the lip traits. (XLSX)

S1 Fig Lip traits. (DOCX)
