# Supplementary material for: An automatic approach for classification and categorisation of lip morphological traits
Source: PLoS One. 2019 Oct 29;14(10):e0221197. doi: 10.1371/journal.pone.0221197 (PMC6818784; doi:10.1371/journal.pone.0221197)
Supplement: S1 Fig — (DOCX) [file pone.0221197.s013.docx]

**Philtrum shape**

| Category number and description | image |
| --- | --- |
| (1) Deep grove,  Philtrum width is narrow | 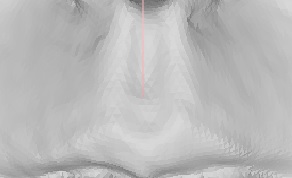 |
| (2) indentation near vermilion border,  narrow philtrum | 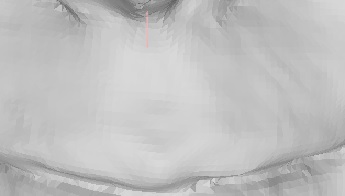 |
| (3) indentation near vermilion border,  wide philtrum | 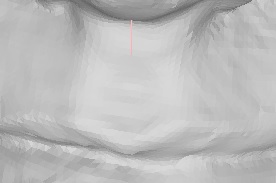 |
| (4) deep groove, average philtrum width | 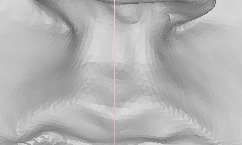 |
| (5 ) no indentation, wide philtrum | 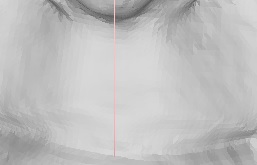 |
| 6 indentation near nose, average philtrum width | 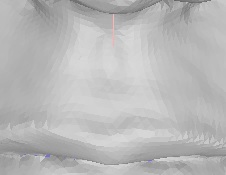 |
| 7 indentation in the middle, average philtrum width | 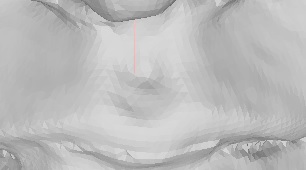 |

**Cupid’s Bow**

| Category number and description | image |
| --- | --- |
| (1) flat | 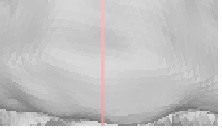 |
| (2) U shape | 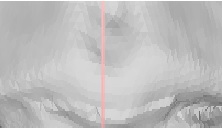 |
| (3) Vshape | 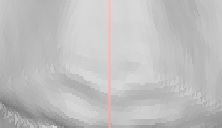 |

**Upper lip contour**

| Category number and description | image |
| --- | --- |
| (1) concave | 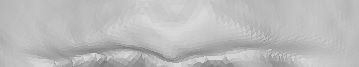 |
| (2) straight | 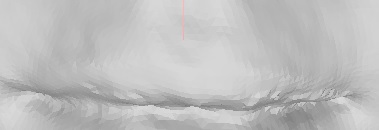 |
| (3) pseudo convex | 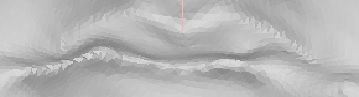 |
| (4) convex | 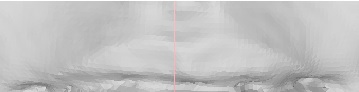 |

**Upper lip border**

| Category number and description | image |
| --- | --- |
| (1 ) full vermilion border and double border | 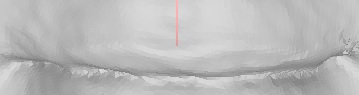 |
| 2 full border with clear boarder at cupid Bow | 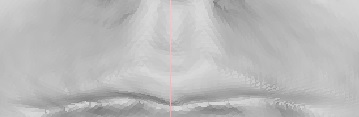 |
| 3 no vermilion boarder | 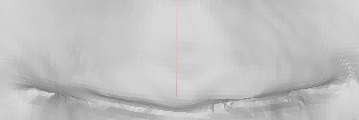 |

**Lower lip contour**

| Category number and description | image |
| --- | --- |
| 1 narrow in the midline | 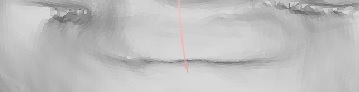 |
| 2 curved | 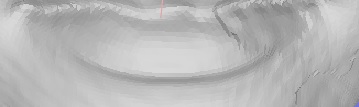 |
| 3 markedly curve | 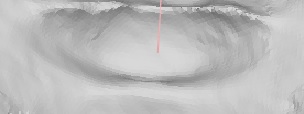 |
| 4 straight | 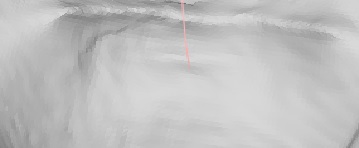 |

**Lower lip border**

| Category number and description | Image |
| --- | --- |
| (1 )non | 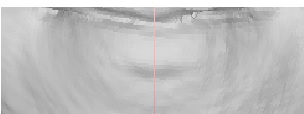 |
| (2)full | 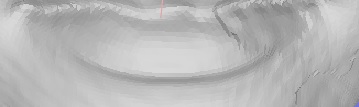 |
| (3) middle | 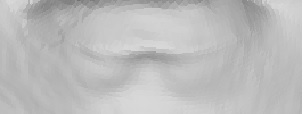 |

**Lip chin profile shape**

| Category number and description |  |
| --- | --- |
| (1) curved concavity | 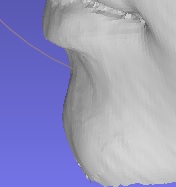 |
| (2) marked angular concavity | 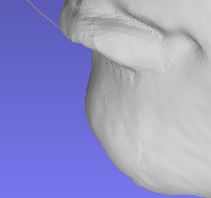 |
| (3) convex area | 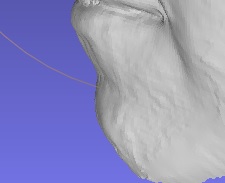 |
| (4) angular concavity | 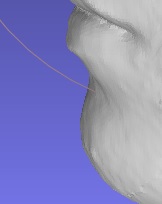 |
| (5) flat | 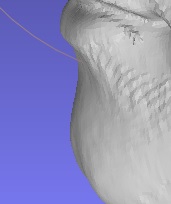 |

**Lower lip Tone**

| Category number and description | image |
| --- | --- |
| (1) none | 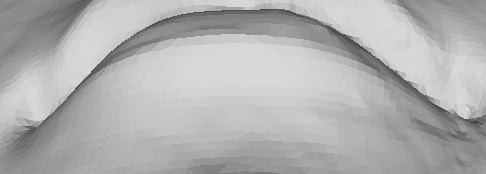 |
| (2) central concavity | 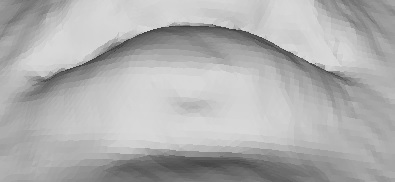 |
| (3) marked lateral mounds and central concavity | 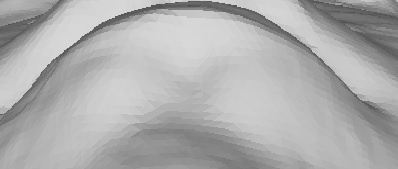 |
| (4) wide concavity | 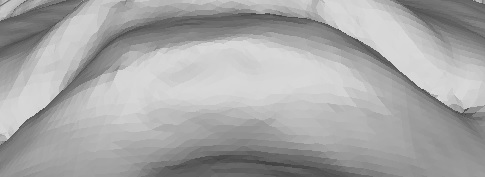 |
| (5) bumped area | 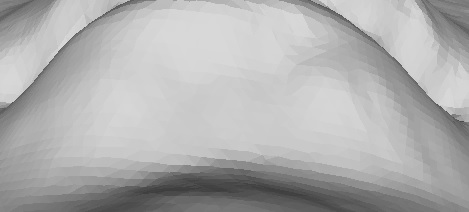 |
